# Elevated Serum Cortisol Levels and associated Factors among Postpartum Mothers in Mbarara district, rural south western Uganda

**DOI:** 10.21203/rs.3.rs-5928934/v1

**Published:** 2025-04-25

**Authors:** Atuhaire Catherine, Taseera Kabanda, Atwine Daniel, Rukundo Zari Godfrey, Byaruhanga Judith, Maling Samuel, Bajunirwe Francis

**Affiliations:** 1 Faculty of medicine, Mbarara University of Science and Technology, Mbarara, Uganda; 2 Soar Research Foundation, PO BOX 1596, Mbarara, Uganda; 3 Department of Psychiatry and Behavioral Neurosciences, McMaster University, Hamilton, Ontario, Canada.; 4 The University of Newcastle, School of Medicine and Public Health, Callaghan, Australia

**Keywords:** Serum cortisol, Postpartum depression, Physical activity, Diabetes mellitus, Rural Uganda

## Abstract

**Background:**

Serum cortisol is often elevated in postpartum mothers, but data on its prevalence and associated factors remain limited in many settings. The current study aimed at examining the factors associated with elevated serum cortisol levels among postpartum mothers in Mbarara district, rural southwestern Uganda.

**Methods:**

We conducted a facility based cross sectional study among mothers between 6 weeks and 6 months after childbirth. Using consecutive sampling, mothers were enrolled from postnatal clinics of two health facilities, Mbarara Regional Referral Hospital and Bwizibwera Health Center IV, a county level health facility in rural southwestern Uganda. Postpartum depression (PPD) was diagnosed using the Mini-International Neuropsychiatric Interview (MINI 7.0.2) for the Diagnostic and Statistical Manual of Mental Disorders, 5^th^ Edition (DSM-5). The blood cortisol levels were measured using a chemiluminiscence with the use of a standard, commercially available competitive immunoassay (Diagnostic Products Corp. Nichols Institute Diagnostics, San Juan Capistrano, CA). The normal range was 5 to 25 mcg/dL.

**Results:**

We enrolled 309 postpartum mothers, and the prevalence of elevated serum cortisol levels was 26.2% (95% CI: 22.0–31.4). Elevated serum cortisol levels were significantly associated with PPD, health facility level attended by the mother, pre-diabetes and diabetes mellitus status, and decreased involvement in physical activity.

**Conclusion:**

In this study of postpartum depression was significantly associated with elevated serum cortisol levels, attending a rural healthcare facility, having pre-diabetes or diabetes mellitus status, and reduced physical activity. These findings underscore the need for targeted holistic interventions addressing both physical and mental health challenges in postpartum women.

## Background to the study

The Postpartum period is of intense vulnerability and stress for the mother and baby (1). It is associated with maternal physical and emotional changes that often increase levels of serum cortisol (2). Cortisol levels remain high in some postpartum women and may not return to pre-pregnant levels until after eight weeks postpartum (3). Cortisol is a glucocorticoid steroid hormone, synthesized from cholesterol in the adrenal cortex and its release is regulated via the hypothalamic pituitary adrenal (HPA) system (4). The HPA system is therefore a key regulating system for stressful and depressive symptoms (5). Cortisol hormone is released into the blood due to stimulation of corticotrophin receptors in the adrenal cortex (6) and serum cortisol levels are important to consider when it comes to a depressed mother (7). Cortisol is the main hormone involved in stress and the fight-or-flight response, a natural and protective response to a perceived threat or danger (8).

A higher than normal level of cortisol may indicate emotional arousal like depression and anxiety (5, 9). In Uganda and other low income settings, maternal mental health is still largely neglected (10). Consequently, there is scanty literature on prevalence of elevated cortisol level and associated factors among postpartum mothers. Yet, elevated serum cortisol levels have been associated with maternal mental blues (11). If maternal mental blues are not identified and managed, the mothers are likely to progress to postpartum depression and its effects. Therefore, in the present study, we assessed the prevalence of elevated serum cortisol levels and associated factors among postpartum mothers in Mbarara district, southwestern Uganda.

## Methods

### Study design and setting

It was a two facility-based cross-sectional study design employing quantitative methods of data collection. This study was conducted in Mbarara Regional Referral Hospital (MRRH) and Bwizibwera health center IV (BHCIV) rural Mbarara district, southwestern Uganda. These health facilities were selected because the majority of deliveries in the district occur there, as they are equipped with fully functional theatres capable of supporting all modes of delivery. MRRH is a public regional referral and teaching hospital for Mbarara University of Science and Technology (MUST) in Mbarara district. It has a bed capacity of 350 and serves a population of over four million people in its catchment area. Every day, over 100 mothers are evaluated at the Maternal and Child Health (MCH) Unit. The MCH has sub-sections of postnatal, young child, family planning, and gynecology clinics. The Postnatal clinic has an average daily attendance of 30 mothers. Approximately 650 postpartum mothers visited MRRH in the previous six months (MRRH records).

BHC IV is a health center that is based in a sub-urban region but largely serves the rural community of Mbarara district. It has an average daily attendance of 14 mothers. Approximately 350 postpartum mothers visited BHC IV in the previous six months (health facility records).

### Study population and period

We enrolled mothers from six weeks to six months’ postpartum as they visited these health facilities. This period was chosen because it coincides with the postnatal review day for both the mother and the child, and it is also a critical time for diagnosing postpartum depression, which is often associated with elevated serum levels. Data were collected from 8^th^ February 2022 to 7^th^ August 2022.

#### Sample size estimation:

The Kish and Leslie formula for calculating sample size to estimate a proportion with a specified margin of error (E) is:

$$N=z^{2}\cdot p\cdot (1-p)/E^{2}$$

Where:

n = required sample size

z = z-value corresponding to the desired confidence level (e.g., 1.96 for a 95% confidence level)

p = prevalence = 16% (12)

E = Desired margin of error (precision) = 0.05

Prevalence (pp) = 16% = 0.16

Confidence Level = 95% (Z-value = 1.96)

$$n=1.96^{2}*0.16(1-0.16)/0.05^{2}$$

n=206.

Considering a design effect of 1.5 to cater for non-random sampling (consecutive), then we used 309 (206*1.5).

#### Sampling procedure:

Using proportionate stratified consecutive sampling method, the sample size was divided proportionately among the two sites with the site as a stratum. Mothers were selected consecutively based on eligibility criteria until the sample size of 309 was attained at the sub-analysis. This was based on the number of women that had given birth in each health facility the previous 6 months (hospital records). 185 mothers were considered for MRRH and 124 for BHC IV.

### Data collection Procedure

All eligible mothers attending any of the study sites provided their written informed consent and were enrolled into the study by the nurses. They were then clinically evaluated by an experienced registered mental health clinician for PPD using the MINI according to the DSM-5 criteria. A pre-coded and pre-tested questionnaire was administered to evaluate for socio-demographic characteristics, clinical signs and symptoms of increased cortisol.

A mother was clinically diagnosed with postpartum depression if she was found to be positive using the Mini-International Neuropsychiatric Interview (MINI 7.0.2) for the Diagnostic and Statistical Manual of Mental Disorders, 5^th^ Edition (DSM-5). The MINI 7.0.2 is a diagnostic interview that follows DSM-5 criteria to determine if a Major Depressive Episode is present in the postpartum period following six weeks. This diagnosis is based on a pattern of symptoms and clinical judgment (13). Mothers suffering from PPD were referred for further management.

### Laboratory assessments

A peripheral blood sample of only 5mls or one teaspoon of blood was drawn from the postpartum mothers between 10.00am and 1.00pm of the same day. In order to avoid the variations of cortisol levels throughout the day, the blood samples were taken at the same time. Plasma cortisol concentrations from all the health facilities were taken to the Mbarara Clinical Research Lab for processing and examination. The blood cortisol levels were measured using a chemiluminiscence with the use of a standard, commercially available competitive immunoassay (Diagnostic Products Corp. or Nichols Advantage, Nichols Institute Diagnostics, San Juan Capistrano, CA). The normal range of serum cortisol among postpartum mothers was noted as 5 to 25 mcg/dL (14).

### Study variables

The main outcome measure was diagnosis of elevated serum cortisol level (>25 mcg/dL) among mothers. This was categorized as a binary variable coded 0=Normal/Low and 1=Elevated.

The independent variables were socio-demographic and medical characteristics and these included:

**Parity** is the number of pregnancies from 28 weeks.

**Residence** was classified as either rural or urban. For this study, urban was defined as people who were residents of Mbarara district with good accessibility to health care, road and commercial settings; while rural areas have a low population density and large amounts of undeveloped land.

**Occupation** was referred to as how people earned a living, ranging from being unemployed to being employed as a professional or laborer according to Uganda Bureau of Statistics.

**Hypertension** was diagnosed when a person’s systolic blood pressure (SBP) was ≥140 mm Hg and/or their diastolic blood pressure (DBP) was ≥90 mm Hg following repeated examination.

**Diabetes Mellitus** diagnosis was based on an *International Classification of Diseases, Ninth Revision (ICD-9)* code by measuring Hemoglobin A1c (HbA1c).

### Quality Control

The PI developed standard operational procedures (SOPs) regarding sample collection, transportation and actual testing of the blood samples. Laboratory tests were done in a quality assured laboratory and validated tools were used. To ensure quality data and data collection procedures, the Principal Investigator (PI) did supportive supervision to ensure that the right tools were used and the sampling procedure to get participants was followed.

#### Data analysis:

The coded data were manually checked, cleaned and double entered into EPI Info software version 7.2. After data entry, appropriate data validation and cleaning was done prior to exporting it to STATA software version 15∙0 (Stata Corp, College Station, TX, USA) for data analysis. Descriptive analysis of participants’ characteristics independent variables [participant’s age, education level, marital status, religion, occupation, cortisol levels etc. and dependent variables] was done. The descriptive statistics were presented as means and standard deviations for continuous variables and frequency and percentages for categorical variables.

Mean cortisol levels were compared using the t-test. Two-tailed t-tests were used to assess the difference in mean values. A significance level of 5% was considered.

Bivariate analysis was done to evaluate the associations between cortisol levels and independent variables to establish their association with cortisol increase and identify possible confounders to adjust in the relationship between cortisol levels. Variables which were found to be statistically significant in the bivariate analysis (associated with *p* value ≤ 0.05), those with p<0.1 and those with biological plausibility (age) with regard to increased cortisol levels were included in the multivariable model building that utilized a manual backward stepwise elimination method. Increased cortisol levels were locked in the model for its effect on PPD to be adjusted for other confounders. All other variables in the final model were reported together with their adjusted OR with the corresponding 95% confidence intervals. A significance level of 5% was considered in this analysis.

## Results

We screened a total of 309 postpartum mothers who visited the postnatal clinic for their 6^th^ week to 6^th^ month postpartum period for eligibility in the study. All the 309 postpartum mothers were enrolled in the study with 185 enrolled at Mbarara Regional Referral Hospital and 124 enrolled at Bwizibwera Health Center IV. The majority were from Mbarara Regional Referral Hospital (MRRH) at 59.9% and resided in urban areas at 57.9%. The study participants had a mean age of 26.7±5.7 years with the majority were under 29 years of age at 67.3%, and had at least a secondary level of education, with 63.8%. The majority were also married, 93.2%, and predominantly Christian at 92.2%. The majority did not have history of alcohol use, 93.5% and had had no physical activity for at least 10 minutes in the past 7 days, 71.2%. In addition, majority of these mothers were 6 – 8 weeks postpartum, 29.4% with the minority at greater than 21 weeks postpartum, 19.7% [Table 1]

Of the 309 postpartum mothers enrolled, the majority of these mothers had 2–4 children, 49%, they were HIV negative, 91.3% and majority reported no/unknown family history of depression, 90.9%, diabetes, 77.3% and hypertension, 68.6%. In addition, majority of the mothers had no hypertension, 62.5% and no DM, 57% [Table 1].

### Prevalence of elevated cortisol levels among postpartum mothers with and without PPD

Of the 309 postpartum mothers enrolled and analyzed, 81 had elevated serum cortisol levels, giving a prevalence of 26.2% (95% CI: 22.0–31.4).

### Factors associated with high serum cortisol levels among postpartum mothers

The sociodemographic factors with an association with elevated serum cortisol levels were: Health Facility, Age category from 29 to 34 years, education of those in secondary school level and above, occupation for those who were civil servants and not engaging in physical activity for at least 10 minutes. Associated medical factors were: being diagnosed with prediabetes, diabetes and PPD. In total, the chi-square bivariate analysis revealed eight factors with statistically significant associations to high serum cortisol levels, p<0.05 [Table 1].

In multivariate analysis, after adjusting for confounders, five variables demonstrated statistically significant predictive values for elevated serum cortisol levels among postpartum mothers (p < 0.05). These included the rural health facility level attended by the mother (AOR = 3.8, 95% CI = 1.9–7.6, p = 0.001), diagnosis with pre-diabetes mellitus (AOR = 2.5, 95% CI = 1.3–5.0, p = 0.008) diabetes mellitus (AOR = 4.0, 95% CI = 1.8–8.9, p = 0.001), and diagnosis with postpartum depression (AOR = 2.9, 95% CI = 1.6–5.3, p < 0.001). Conversely, involvement in physical activity was associated with a decreased likelihood of elevated serum cortisol levels (AOR = 0.3, 95% CI = 0.1–0.7, p = 0.002) [Table 2].

## Discussion

This study aimed at determining the prevalence of elevated serum cortisol levels and its associated factors among postpartum mothers. Overall, we noted a prevalence of 26.2%. Factors associated with elevated serum cortisol were: health facility of attendance, being a pre-diabetic or diabetic, diagnosis with postpartum depression and involvement in physical activity.

### Prevalence of elevated serum cortisol levels among postpartum mothers

The prevalence of elevated serum cortisol in our study was higher than serum cortisol levels of postpartum mothers reported in Sweden (18%). Cortisol is a stress hormone, and its levels are naturally elevated in response to stress. Uganda is a low-income country which often experiences higher levels of poverty, food insecurity, lack of access to quality healthcare, and housing instability, which contribute to psychosocial stress or postpartum depression. Postpartum mothers in Uganda may also face greater financial burdens, caregiving responsibilities, and limited maternity care services, all of which can elevate cortisol levels. This also aligns with previous studies which reported that dysregulation of the HPA axis is evident in postpartum women experiencing elevated serum cortisol levels and this may be due to the activation of the HPA axis in response to stress (11, 15). Our study’s findings underscore the need for routine screening of cortisol levels, especially in settings where maternal mental health resources may be limited. Early identification of elevated cortisol could enable timely intervention and improve maternal health outcomes.

### Factors associated with high serum cortisol levels among postpartum mothers

Our analysis identified five key factors significantly associated with elevated serum cortisol levels in postpartum mothers. These included suffering from postpartum depression, the rural health facility level attended by the mother, diagnosis with pre-diabetes and diabetes mellitus status, and decreased involvement in physical activity.

Mothers who were suffering from PPD in the present study, were found to be 2.9 times more likely to have elevated serum cortisol levels than mothers without PPD. The association between PPD and elevated serum cortisol levels has not been adequately studied in low-resourced settings but this significant association underscores the impact of PPD on the physiological stress response. In mothers with PPD, the body’s stress response system is often dysregulated, leading to elevated levels of cortisol. These findings corroborate previous studies, such as those by (5, 15), which demonstrated the link between HPA axis dysregulation and depressive symptoms as discussed above. It is important to note that these mothers’ feedback mechanisms that usually normalize cortisol levels are often impaired.

The health facility may predict elevated serum cortisol levels of postpartum mothers. From this study, mothers attending Bwizibwera Health Center IV had 2.7 times higher odds of elevated cortisol levels compared to those attending Mbarara Regional Referral Hospital. This might reflect differences in the settings of these health facilities, patient populations or the quality of care provided within these facilities. BHC IV serves a rural population and mothers in the rural settings may exacerbate raised serum cortisol levels due to the quality of care in this facility. Several studies from middle income countries, such as Bangladesh, India, Iran and Pakistan concur with our findings that elevated serum cortisol levels are more prevalent in mothers residing in rural settings and have poor access to health facilities in the rural settings (16–20). In a study where Rahman examined the quality of healthcare and patient satisfaction across different levels of health facilities, he highlights that patient satisfaction is correlated with the quality of care provided, which can vary meaningfully between different levels of health facilities (20). Rural health facilities often have fewer resources and staff, which can lead to delays in care and increased serum cortisol levels for postpartum mothers. In addition, mothers in the rural settings commonly depend on subsistence agriculture with low standards of living and have financial difficulties than women in urban settings (21).

Our study revealed that Diabetes Mellitus was high among mothers with elevated serum cortisol levels. Diabetic postpartum mothers were 4 times more likely to have raised serum cortisol levels. This finding was congruent with similar studies that suggested a possible link between insulin resistance and the features of metabolic syndrome (22). Cortisol is a steroid hormone that plays a crucial role in the body’s stress response and helps regulate various physiological processes, including metabolism. While cortisol is essential for normal bodily functions, chronically elevated levels are often associated with chronic stress or PPD. Elevated cortisol levels can worsen insulin resistance hence diabetes mellitus and this creates a feedback loop that contributes to higher cortisol levels. Diabetes can affect the HPA axis, which regulates cortisol production which in turn leads to altered cortisol secretion patterns and elevated baseline levels (4).

Finally, decreased involvement in physical activity for at least ten minutes as recommended for postpartum mothers was significantly associated with elevated cortisol levels. Physical activity is known to reduce stress by promoting the release of endorphins and other neurotransmitters that improve mood. Without regular physical activity for at least ten minutes, postpartum mothers may experience higher levels of stress, which can lead to increased cortisol production. Furthermore, lack of physical activity can lead to dysregulation of the HPA axis, resulting in elevated cortisol levels (4). In addition, physical activity is associated with weight management for postpartum mothers (23) but interestingly, this study documented no significant correlation between BMI and elevated cortisol levels.

## Conclusions

This study found that 26.2% of postpartum mothers in Mbarara District, rural southwestern Uganda had elevated serum cortisol levels, with significant associations identified between elevated cortisol, diagnosis of postpartum depression, rural healthcare facility level, pre-diabetes/diabetes status, and reduced physical activity. This study highlights the need for routine cortisol screening and targeted interventions to manage stress and improve maternal health outcomes.

### Policy implications

Policy should integrate routine cortisol screening among postpartum care to reduce stress-related health risks. Elevated cortisol levels may serve as a biomarker for identifying mothers at risk of developing postpartum depression and other related health issues. Policy should also advocate for physical activity promotion for at least ten minutes among postpartum mothers. Improving healthcare access and resources in rural areas is crucial to mitigating cortisol dysregulation.

## Figures and Tables

**Table 1.** Participants’ characteristics and Results of Bivariate analysis for factors associated with elevated serum cortisol levels among postpartum mothers

Factors		Overall, n (%)	Normal/low, n (%)	Elevated, n (%)	UOR (95% CI)	p-value
Facility	MRRH	**185 (59.9)**	146 (78.9)	39 (21.1)	1	
	BHCIV	124 (40.1)	82 (66.1)	42 (33.9)	1.9 (1.1 – 3.2)	0.018*
Residence	Rural	130 (42.1)	91 (70.0)	39 (30.0)	1	
	Urban	**179 (57.9)**	137 (76.5)	42 (23.5)	0.7 (0.4 – 1.2)	0.201
Age	<29 years	**208 (67.3)**	158 (76.0)	50 (24.0)	1	
	29–34 years	65 (21.0)	40 (61.5)	25 (38.5)	2.0 (1.1 – 3.6)	0.023*
	>34 years	36 (11.7)	30 (83.3)	6 (16.7)	0.6 (0.2 – 1.6)	0.304
Education	<secondary	112 (36.2)	91 (81.3)	21 (18.8)	1	
	≥Secondary	**197 (63.8)**	137 (69.5)	60 (30.5)	1.9 (1.1 – 3.3)	0.026*
Marital Status	Unmarried	21 (6.8)	16 (76.2)	5 (23.8)	1	
	Married	**288 (93.2)**	212 (73.6)	76 (26.4)	1.1 (0.4 – 3.2)	0.776
Tribe	Munyankore	**242 (78.3)**	173 (71.5)	69 (28.5)	1	
	Muganda	17 (5.5)	15 (88.2)	2 (11.8)	0.3 (0.1 – 1.5)	0.156
	Mukiga	30 (9.7)	25 (83.3)	5 (16.7)	0.5 (0.2 – 1.4)	0.171
	Others	20 (6.5)	15 (75.0)	5 (25.0)	0.8 (0.3 – 2.4)	0.738
Occupation	Housewife	77 (24.9)	57 (74.0)	20 (26.0)	1	
	Business	126 (40.8)	102 (81.0)	24 (19.0)	0.7 (0.3 – 1.3)	0.269
	Peasant	65 (21.0)	46 (70.8)	19 (29.2)	1.2 (0.6 – 2.5)	0.660
	Civil Servant	41 (13.3)	23 (56.1)	18 (43.9)	2.2 (1.0 – 5.0)	0.050*
Religion	Christians	**285 (92.2)**	208 (73.0)	77 (27.0)	1	
	Muslims	24 (7.8)	20 (83.3)	4 (16.7)	0.5 (0.2 – 1.6)	0.280
Alcohol Consumption	No	289 (93.5)	217 (75.1)	72 (24.9)	1	
	Yes	20 (6.5)	11 (55.0)	9 (45.0)	2.5 (1.0 – 6.2)	0.057
Physical Activity of <10 minutes in past 7 days	Yes	89 (28.8)	74 (83.2)	15 (16.8)	1	
	No	**220 (71.2)**	154 (70.0)	66 (30.0)	2.1 (1.1 – 4.0)	0.018*
Postpartum period (Weeks)	6–8 weeks	**91 (29.4)**	71 (78.0)	20 (22.0)	1	
	9–12 weeks	70 (22.7)	47 (67.1)	23 (32.9)	1.7 (0.9 – 3.5)	0.137
	13–20 weeks	87 (28.2)	66 (75.9)	21 (24.1)	1.1 (0.6 – 2.3)	0.723
	≥21 weeks	61 (19.7)	44 (72.1)	17 (27.9)	1.4 (0.6 – 2.9)	0.421
Parity	Primigravida	133 (43.0)	102 (76.7)	31 (23.3)	1	
	Multipara	**152 (49.2)**	112 (73.7)	40 (26.3)	1.2 (0.7 – 2.0)	0.570
	Grandmultipara	24 (7.8)	14 (58.3)	10 (41.7)	2.4 (1.0 – 5.8)	0.065
HIV Serostatus	Positive	27 (8.7)	21 (77.8)	6 (22.2)	1	
	Negative	**282 (91.3)**	207 (73.4)	75 (26.6)	1.3 (0.5 – 3.3)	0.640
Family History of Depression	No/DK	**281 (90.9)**	209 (74.4)	72 (25.6)	1	
	Yes	28 (9.1)	19 (67.9)	9 (32.1)	1.4 (0.6 – 3.2)	0.457
Family History of Diabetes	No/DK	**239 (77.3)**	176 (73.6)	63 (26.4)	1	
	Yes	70 (22.7)	52 (74.3)	18 (25.7)	1.0 (0.5 – 1.8)	0.918
Family History of Hypertension	No/DK	**212 (68.6)**	155 (73.1)	57 (26.9)	1	
	Yes	97 (31.4)	73 (75.3)	24 (24.7)	0.9 (0.5 – 1.6)	0.686
Hypertension	No	**193 (62.5)**	139 (72.0)	54 (28.0)	1	
	Yes	116 (37.5)	89 (76.7)	27 (23.3)	1.3 (0.7 – 2.2)	0.401
Body Mass Index (Kg/m^2^)	Normal	177 (57.3)	133 (75.1)	44 (24.9)	1	
	Underweight	16 (5.2)	14 (87.5)	2 (12.5)	2.3 (0.6 – 8.6)	0.233
	Overweight	81 (26.2)	58 (71.6)	23 (28.4)	0.9 (0.5 – 1.5)	0.633
	Obese	35 (11.3)	23 (65.7)	12 (34.3)	1.8 (0.8 – 4.1)	0.185
Pulse rate (bpm)	Normal	292 (94.8)	214 (73.3)	78 (26.7)	1	
	Low	4 (1.3)	3 (75.0)	1 (25.0)	0.8 (0.1 – 7.2)	0.862
	High	12 (3.9)	10 (83.3)	2 (16.7)	0.6 (0.2 – 2.2)	0.433
Diabetes Mellitus	Normal	**176 (57.0)**	143 (81.3)	33 (18.8)	1	
	Pre-Diabetes Mellitus	78 (25.2)	52 (66.7)	26 (33.3)	2.2 (1.2 – 4.0)	0.012*
	Diabetes Mellitus	55 (17.8)	33 (60.0)	22 (40.0)	2.9 (1.5 – 5.6)	0.002**
Postpartum depression	No	184 (59.5)	151 (82.1)	33 (17.9)	1	
	Yes	125 (40.5)	77(61.6)	48(38.4)	2.9 (1.7 – 4.8)	<0.001***

* DK = don’t know

* p < 0.05,

** p < 0.01,

*** p < 0.001

MRRH: Mbarara Regional Referral Hospital, BHC IV: Bwizibwera Health Center IV, HIV: Human Immunodeficiency Virus, UOR: Unadjusted Odds Ratio

**Table 2.** Results of multivariate analysis for factors associated with high serum cortisol levels among postpartum mothers

Factors	Category	AOR (95% CI)	p-value
Facility	MRRH	1	
	BHCIV	3.8 (1.9 – 7.6)	<0.001***
Age	<29 years	1	
	29–34 years	1.5 (0.8 – 3.0)	0.248
	>34 years	0.3 (0.1 – 1.0)	0.052
Education	<Secondary	1	
	≥Secondary	1.3 (0.7 – 2.6)	0.386
Occupation	Housewife	1	
	Business	0.5 (0.2 – 1.1)	0.076
	Peasant	1.0 (0.4 – 2.7)	0.920
	Civil Servant	2.0 (0.8 – 5.0)	0.156
Physical Activity of <10 minutes in past 7 days	No	1	
	Yes	0.3 (0.1 – 0.7)	0.002**
Diabetes Mellitus	Normal	1	
	Pre-Diabetes Mellitus	2.5 (1.3 – 5.0)	0.008**
	Diabetes Mellitus	4.0 (1.8 – 8.9)	0.001**
Postpartum depression	No	1	
	Yes	2.9 (1.6 – 5.3)	<0.001***

* p < 0.05,

** p < 0.01,

*** p < 0.001

MRRH: Mbarara Regional Referral Hospital, BHC IV: Bwizibwera Health Center IV, HIV: Human Immunodeficiency Virus, AOR: Adjusted Odds Ratio

## Data Availability

All relevant data are included in the manuscript and all the data underlying the study findings will be available without restrictions if requested.

## References

[1] Davis EP, Narayan AJ Journal of Abnormal Psychology, psychopathology. Pregnancy as a period of risk, adaptation, and resilience for mothers and infants. 2020;32(5):1625–39. 10.1017/S0954579420001121 PMC7863987 33427164

[2] Seth S, Lewis AJ, Galbally M Journal of Affective Disorders, childbirth. Perinatal maternal depression and cortisol function in pregnancy and the postpartum period: a systematic literature review. 2016;16:1–19. 10.1186/s12884-016-0915-y PMC4886446 27245670

[3] Osayande OE, Ekpruke CD, Inikogbashe D African Scientist. Serum Cortisol Levels in Pregnancy and Six Weeks Post-Partum. 2017;18:235–8.

[4] Tsigos C, Chrousos GP Journal of Psychosomatic Research. Hypothalamic–pituitary–adrenal axis, neuroendocrine factors and stress. 2002;53(4):865–71. 10.1016/s0022-3999(02)00429-4 12377295

[5] Islam MR, Islam MR, Ahmed I, Moktadir AA, Nahar Z, Islam MS, Journal of Depression and Anxiety. Elevated serum levels of malondialdehyde and cortisol are associated with major depressive disorder: a case-control study. 2018;6:2050312118773953. 10.1177/2050312118773953 PMC5946642 29770218

[6] Leibowitz A, Boyko M, Shapira Y, Zlotnik A International Journal of Molecular Sciences. Blood glutamate scavenging: insight into neuroprotection. 2012;13(8):10041–66. 10.3390/ijms130810041 PMC3431845 22949847

[7] Stephens MAC, Mahon PB, McCaul ME, Wand GS Psychoneuroendocrinology. Hypothalamic–pituitary–adrenal axis response to acute psychosocial stress: Effects of biological sex and circulating sex hormones. 2016;66:47–55. 10.1016/j.psyneuen.2015.12.021 PMC4788592 26773400

[8] Russell G, Lightman S Neurobiology of Stress. The human stress response. 2019;15(9):525–34. 10.1038/s41574-019-0228-0 31249398

[9] O’neill P, Davies I, Fullerton K, Bennett D Journal of the Neurological Sciences. Stress hormone and blood glucose response following acute stroke in the elderly. 1991;22(7):842–7. 10.1161/01.str.22.7.842 1853403

[10] Ashaba S, Rukundo GZ, Beinempaka F, Ntaro M, LeBlanc JC BMC Public Health. Maternal depression and malnutrition in children in southwest Uganda: a case control study. 2015;15:1–6. 10.1186/s12889-015-2644-y PMC4693407 26712120

[11] Osayande OE, Ekpruke CD, Inikogbashe D. Serum Cortisol Levels in Pregnancy and Six Weeks Post-Partum. African Scientist. 2017;18:235–8.

[12] Khan KS, Wojdyla D, Say L, Gülmezoglu AM, Van Look PF The Lancet. WHO analysis of causes of maternal death: a systematic review. 2006;367(9516):1066–74. 10.1016/S0140-6736(06)68397-9 16581405

[13] Segre LS, O’Hara MW, Arndt S, Stuart S Social Psychiatry and Psychiatric Epidemiology, epidemiology p. The prevalence of postpartum depression: the relative significance of three social status indices. 2007;42:316–21. 10.1007/s00127-007-0168-1 17370048

[14] Sapse AT Psychoneuroendocrinology. Cortisol, high cortisol diseases and anti-cortisol therapy. 1997;22:S3–S10. 10.1016/s0306-4530(97)00024-3 9264141

[15] Glynn LM, Davis EP, Sandman CA Neurobiology of Depression. New insights into the role of perinatal HPA-axis dysregulation in postpartum depression. 2013;47(6):363–70. 10.1016/j.npep.2013.10.007 24210135

[16] Villegas L, McKay K, Dennis CL, Ross LEJ The Journal of Rural Health. Postpartum depression among rural women from developed and developing countries: a systematic review. 2011;27(3):278–88. 10.1111/j.1748-0361.2010.00339.x 21729155

[17] Gausia K, Fisher C, Ali M, Oosthuizen J Psychological Medicine. Magnitude and contributory factors of postnatal depression: a community-based cohort study from a rural subdistrict of Bangladesh. 2009;39(6):999–1007. 10.1017/S0033291708004455 18812008

[18] Savarimuthu RJ, Ezhilarasu P, Charles H, Antonisamy B, Kurian S, Jacob KS Indian J Soc Psychiatry. Post-partum depression in the community: a qualitative study from rural South India. 2010;56(1):94–102. 10.1177/0020764008097756 19906768

[19] Goker A, Yanikkerem E, Demet MM, Dikayak S, Yildirim Y, Koyuncu FM ISRN Obstetrics and Gynecology. Postpartum depression: is mode of delivery a risk factor? 2012;2012(1):616759. 10.5402/2012/616759 PMC3530850 23304542

[20] Rahman A, Iqbal Z, Harrington R Psychological Medicine. Life events, social support and depression in childbirth: perspectives from a rural community in the developing world. 2003;33(7):1161–7. 10.1017/s0033291703008286 14580070

[21] UBoS ICF International, Uganda. Uganda demographic and health survey 2016: key indicators report. 2017.

[22] Andrews RC, Herlihy O, Livingstone DE, Andrew R, Walker BR, J Clin Endocrinol Metab. Abnormal cortisol metabolism and tissue sensitivity to cortisol in patients with glucose intolerance. 2002;87(12):5587–93. 10.1210/jc.2002-020048 12466357

[23] Kaur D, Malhotra A, Ranjan P, Chopra S, Kumari A, Vikram NK, Diabetes & Metabolic Syndrome: Clinical Research & Reviews Weight management in postpartum women-An Indian perspective. 2021;15(6):102291. 10.1016/j.dsx.2021.102291 34598009

